# Physiological and Biochemical Aspects of Silicon-Mediated Resistance in Maize against Maydis Leaf Blight

**DOI:** 10.3390/plants13040531

**Published:** 2024-02-15

**Authors:** Luis Felipe Lata-Tenesaca, Marcos José Barbosa Oliveira, Aline Vieira Barros, Bárbara Bezerra Menezes Picanço, Fabrício Ávila Rodrigues

**Affiliations:** Departamento de Fitopatologia, Laboratório da Interação Planta-Patógeno, Universidade Federal de Viçosa, Viçosa, Minas Gerais 36570-900, Brazil; lfelipelata@gmail.com (L.F.L.-T.); marcos.j.oliveira@ufv.br (M.J.B.O.); alinevieiradebarros@gmail.com (A.V.B.); barbara.picanco@gmail.com (B.B.M.P.)

**Keywords:** antioxidative enzymes, defense enzymes, host defense reactions, fluorescence of chlorophyll *a*, photosynthesis, X-ray microanalysis

## Abstract

Maydis leaf blight (MLB), caused by the necrotrophic fungus *Bipolaris maydis*, has caused considerable yield losses in maize production. The hypothesis that maize plants with higher foliar silicon (Si) concentration can be more resistant against MLB was investigated in this study. This goal was achieved through an in-depth analysis of the photosynthetic apparatus (parameters of leaf gas exchange chlorophyll (Chl) *a* fluorescence and photosynthetic pigments) changes in activities of defense and antioxidative enzymes in leaves of maize plants with (+Si; 2 mM) and without (−Si; 0 mM) Si supplied, as well as challenged and not with *B. maydis*. The +Si plants showed reduced MLB symptoms (smaller lesions and lower disease severity) due to higher foliar Si concentration and less production of malondialdehyde, hydrogen peroxide, and radical anion superoxide compared to −Si plants. Higher values for leaf gas exchange (rate of net CO_2_ assimilation, stomatal conductance to water vapor, and transpiration rate) and Chl *a* fluorescence (variable-to-maximum Chl *a* fluorescence ratio, photochemical yield, and yield for dissipation by downregulation) parameters along with preserved pool of chlorophyll *a*+*b* and carotenoids were noticed for infected +Si plants compared to infected −Si plants. Activities of defense (chitinase, β-1,3-glucanase, phenylalanine ammonia-lyase, polyphenoloxidase, peroxidase, and lipoxygenase) and antioxidative (ascorbate peroxidase, catalase, superoxide dismutase, and glutathione reductase) enzymes were higher for infected +Si plants compared to infected −Si plants. Collectively, this study highlights the importance of using Si to boost maize resistance against MLB considering the more operative defense reactions and the robustness of the antioxidative metabolism of plants along with the preservation of their photosynthetic apparatus.

## 1. Introduction

Maize (*Zea mays* L.) production plays a crucial role in food security, economic development, and environmental sustainability in many countries for being part of the human diet and with various industrial applications [1]. Maize crop is affected by several diseases caused by pathogens of different lifestyles that significantly contribute to decreased grain quality and yield [2]. Among the diseases affecting maize production worldwide, maydis leaf blight (MLB), caused by the fungus *Bipolaris maydis* (Y. Nisik. & C. Miyake) Shoemaker, has become of economic importance [3]. It is a widespread foliar disease and represents a potential threat to global maize production mainly in developing countries [4,5].

During the infection process of *B. maydis*, the germ tubes formed from a germinated conidium penetrate the leaves through stomata or directly through the cuticular walls with the aid of hydrolytic enzymes [6]. The major MLB symptoms are rectangular to elliptical necrotic lesions of brown to tan colors that develop on both abaxial and adaxial leaf surfaces [7]. Yield losses caused by MLB are greatly associated with damage to the photosynthetic apparatus due to an extensive leaf area with larger necrotic lesions surrounded by intense chlorosis, reduced plant growth, and poor allocation of assimilates from leaves to the developing grains [4]. The management of MLB has been achieved using maize genotypes with high basal levels of resistance [8]. Foliar sprays of different fungicides and crop rotation are key strategies used for MLB control [9,10]. The disease epidemic rate can be slowed by keeping adequate levels of macro and micronutrients in the plant tissues during the different plant growth stages [11].

Silicon (Si), the second most abundant element in nature after oxygen, allows plants to perform well when facing various types of abiotic and biotic stresses [12,13]. The benefits obtained with Si fertilization were mainly overlooked until the beginning of the 20th century, partly due to its abundance in nature besides the lack of visible symptoms of either deficiency or toxicity [14]. The Si is absorbed from the soil solution in the form of monosilicic acid (H_4_SiO_4_) and transported to shoots [12]. The passive mechanism of Si uptake by plants occurs via mass flow [12]. Particularly in maize, plants can actively uptake and accumulate high amounts of Si on leaves due to the expression of *ZmLsi1*, *ZmLsi2*, and *ZmLsi6* genes [15,16]. The proteins originated from *ZmLsi1* and *ZmLsi2* genes allow the H_4_SiO_4_ to pass through the root cells to shoot via transpiratory flow, and the discharge into the xylem vessels occurs through the expression of *ZmLsi6* [15,16].

The effect of Si on plant–pathogen interactions has centered around two main mechanisms known as physical and or biochemical/molecular [12,13,14]. The physical role of Si is due to the accumulation of this element below the cuticle and in the cell wall of epidermal cells, which acts as a stronger physical barrier (polymerized H_4_SiO_4_) that may prevent or delay the penetration of some pathogens [12,13]. The biochemical resistance mediated by Si is associated with the potentiation of defense-related enzymes (e.g., chitinases, *β*-1,3-glucanase, phenylalanine ammonia-lyase, polyphenoloxidase, and peroxidase) and metabolites (e.g., dopamine, phenolics compounds, flavonoids, phytoalexin, and lignin), and a more robust antioxidative metabolism [12,13,17]. Physiological processes enhanced by Si are related to the improved photosynthetic performance of stressed plants by pathogen infection and their carbohydrate metabolism regulation [13]. The intensities of some diseases such as smut (*Ustilago maydis*), macrospora leaf spot (*Stenocarpella macrospora*), pythium root rot (*Pythium aphanidermatum*), and stalk rot (*Fusarium graminearum* and *Fusarium moniliforme*) were significantly reduced by supplying Si to maize plants [12,13,14,17].

The photosynthetic parameters obtained by performing leaf gas exchange and chlorophyll *a* fluorescence measurements in the different organs of plants (e.g., roots, vascular system, leaves, and fruits) facing infection by pathogens of different lifestyles are good physiological indicators of their prompt defense against them [17,18]. Generally, plants supplied with Si display better photosynthetic performance and maintain the pool of photosynthetic pigments [18]. For instance, maize plants supplied with Si showed more operative photosynthetic machinery and more efficient antioxidant metabolism during infection by *Exserohilum turcicum* and *Stenocarpella macrospora* [19,20].

Considering the mechanistic basis behind the potentiation of resistance of different profitable crops against diseases mediated by Si and the lack of information on the physiological and biochemical process taking place in the maize-*B. maydis* interaction, the present study hypothesized that Si could allow maize plants to respond against fungal infection more efficiently. It is expected that maize plants having higher foliar Si concentration will show earlier and stronger defense reactions as well as better photosynthetic performance in combination with the well-coordinated and efficient regulation of carbohydrate metabolism and more robust antioxidant metabolism to minimize the damage caused by *B. maydis* infection.

## 2. Results

### 2.1. Analysis of Variance

The factor Si rates was significant for severity, *A*, *F*_v_/*F*_m_, Y(II), ETR, MDA, O_2_^•−^, PAL, and LTGA derivatives. The factor plant inoculation was significant for *A*, *F*_v_/*F*_m_, Y(NPQ), Chl *a*+*b*, Car, glucose, sucrose, starch, MDA, O_2_^•−^, TSP, LTGA derivatives, CHI, GLU, PAL, PPO, POX, LOX, SOD, APX, and CAT. The interaction Si rates × plant inoculation was significant for *F*_v_/*F*_m_, sucrose, MDA, O_2_^•−^, and LTGA derivatives (Table 1).

### 2.2. Foliar Si and K Concentrations and X-ray Microanalysis

**Figure 1:**
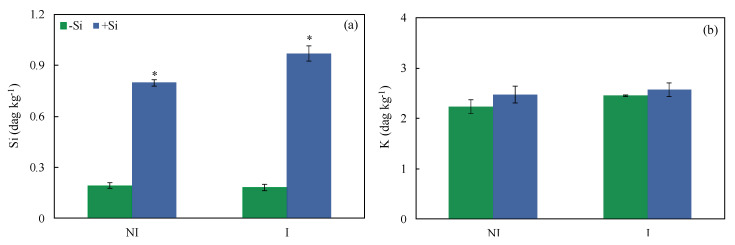
Foliar concentrations of silicon (Si) (**a**) and potassium (K) (**b**) for maize plants non-supplied (−Si) or supplied (+Si) with silicon (Si) and non-inoculated (NI) or inoculated (I) with *Bipolaris maydis*. Means for −Si and +Si treatments for NI and I plants followed by an asterisk (*) are significantly different (*p* ≤ 0.05) according to the *F* test. Bars represent the standard error of the means.

**Figure 2:**
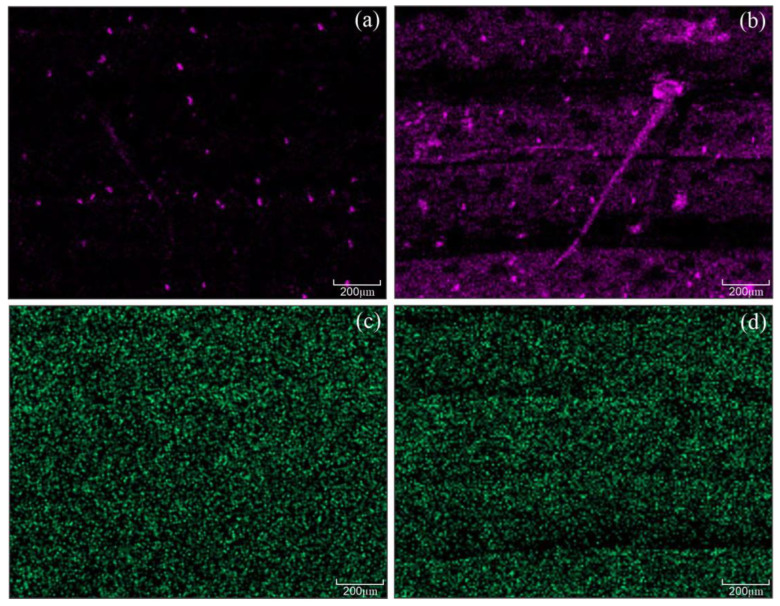
X-ray maps for the presence of silicon (Si) (**a**,**b**) and potassium (K) (**c**,**d**) in the leaves of maize plants non-supplied (−Si) (a and c) or supplied (+Si) (b and d) with Si at 156 h after inoculation with *Bipolaris maydis* (isolate UFV-DPF-*Bm*12). In the X-ray maps, purple (**a**,**b**) and green (**c**,**d**) fluorescent dots correspond to the accumulation of Si or K, respectively. Black indicates the absence of Si or K. Scale bars = 200 μm.

At the end of the experiment (156 hai), the fifth and sixth leaves of each plant per replication of each treatment that was grown under greenhouse conditions were collected to determine the foliar concentrations of Si and K. Foliar Si concentration significantly increased by 70 and 72% for non-inoculated +Si plants and inoculated +Si plants, respectively, compared to −Si treatments (Figure 1a). There was no significant difference between −Si and +Si plants, regardless of plant inoculation with *B. maydis* for foliar K concentration (Figure 1b). In order to gain more information regarding the presence of silicon in the leaf tissues of −Si and +Si plants infected by *B. maydis*, leaf fragments were processed for X-ray microanalysis. This analysis indicated a differential pattern of Si deposition on the adaxial surface of leaves from −Si and +Si plants at 156 hai (Figure 2). The intensity of Si and K depositions were obtained based on the X-ray emission spectra of the X-ray maps [Si = 0.81, error (E) = 0.04 (a); Si = 8.35, E = 0.09 (b); K = 5.91, E = 0.09 (c); and K = 4.25, E = 0.07 133 (d)] compared to other chemical elements such as carbon, oxygen, magnesium, phosphorus, sulfur, and chlorine. The levels of Si deposition in the infected leaves of +Si plants were 90% higher (8.35 wt %) compared to −Si plants (0.81 wt %) (Figure 2a,b). The K deposition was similar between the adaxial surface of leaves of −Si (5.91 wt %) and +Si (4.25 wt %) plants (Figure 2c,d).

### 2.3. Symptoms of MLB and Severity

**Figure 3:**
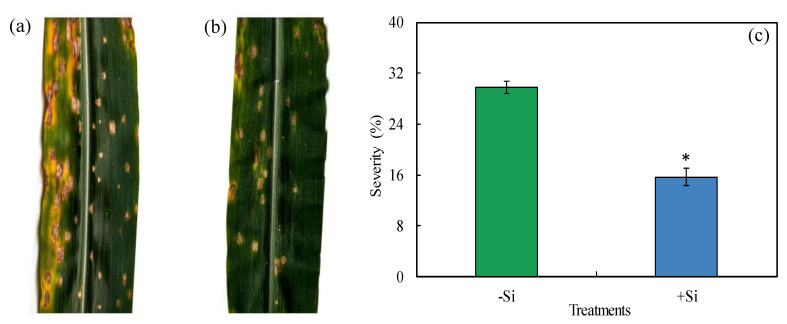
Symptoms of maydis leaf blight (**a**,**b**) and severity (**c**) for maize plants non-supplied (−Si) or supplied (+Si) with silicon (Si). Means for −Si and +Si treatments (graph **c**) followed by an asterisk (*) are significantly different (*p* ≤ 0.05) according to the *F* test. Bars represent the standard error of the means. Imaging of leaves with disease symptoms and the assessment of severity were performed at 156 h after plant inoculation with *Bipolaris maydis* (isolate UFV-DPF-*Bm*12).

The leaves from −Si and +Si plants at the V6 growth stage were inoculated with a conidial suspension of *B. maydis* (isolate UFV-DPF-*Bm*12). At 156 hai, the symptoms of maydis leaf spot and its severity on the fifth expanded leaf were evaluated. In the leaves of −Si plants, disease symptoms (number and size of the necrotic lesions, lesions coalescence, and chlorosis) were remarkably more expressive than in the leaves of +Si plants (Figure 3a,b). The severity of MLB was significantly reduced by 47% for +Si plants compared to −Si plants (Figure 3c).

### 2.4. Leaf Gas Exchange Parameters

**Figure 4:**
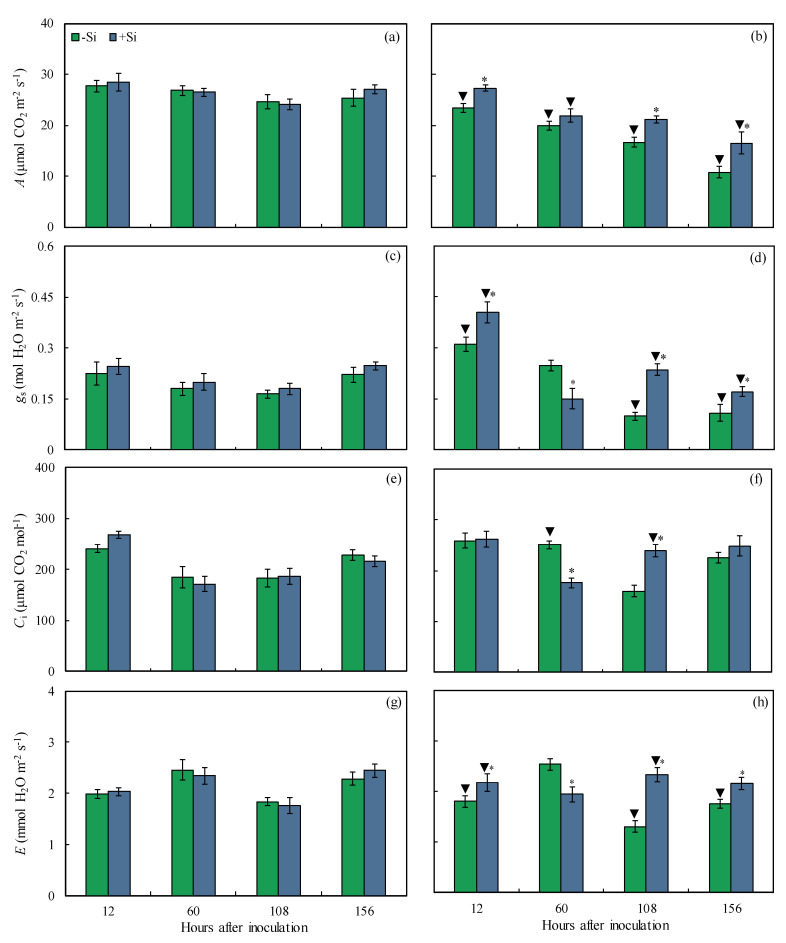
Leaf gas exchange parameters net carbon assimilation rate (*A*) (**a**,**b**), stomatal conductance to water vapor (*g*_s_) (**c**,**d**), internal CO_2_ concentration (*C*_i_) (**e**,**f**), and transpiration rate (*E*) (**g**,**h**) determined on the leaves of maize plants non-inoculated (NI) (**a**,**c**,**e**,**g**) or inoculated (I) (**b**,**d**,**f**,**h**) with *Bipolaris maydis* (isolate UFV-DPF-*Bm*12) and non-supplied (−Si) or supplied (+Si) with silicon (Si). Means for NI and I plants followed by an inverted triangle (▼) and for −Si and +Si treatments followed by an asterisk (*), at each evaluation time, are significantly different (*p* ≤ 0.05) according to the *F* test. Bars represent the standard error of the means.

The values of leaf gas exchange parameters were obtained from non-inoculated and inoculated plants that were non-supplied or supplied with Si from 12 to 156 hai. The leaf gas exchange parameters will be good physiological indicators of the robustness of the photosynthetic apparatus of +Si-infected plants in comparison to −Si-infected plants. For inoculated −Si plants, *A* (15, 21, and 35% at 12, 108, and 156 hai, respectively), *g*_s_ (23, 57, and 37% at 12, 108, and 156 hai, respectively), and *E* (17, 44, and 19% at 12, 108, and 156 hai, respectively) were significantly lower while *g*_s_, *C*_i_, and *E* (39, 29, and 23% at 60 hai, respectively) were significantly higher compared to inoculated +Si plants (Figure 4b,d,f,h). The *A* (15–58% from 12 to 156 hai), *g*_s_ (39 and 51% at 108 and 156 hai, respectively), and *E* (10, 29, and 23% at 12, 108, and 156 hai, respectively) were significantly lower for inoculated −Si plants compared to non-inoculated −Si plants (Figure 4a–d,g–h). Significant and lower values for *A* (17 and 39% at 60 and 156 hai, respectively) and *g*_s_ (30% at 156 hai) occurred for inoculated +Si plants compared to non-inoculated +Si plants (Figure 4a–d).

### 2.5. Imaging and Quantification of Chl a Fluorescence Parameters

**Figure 5:**
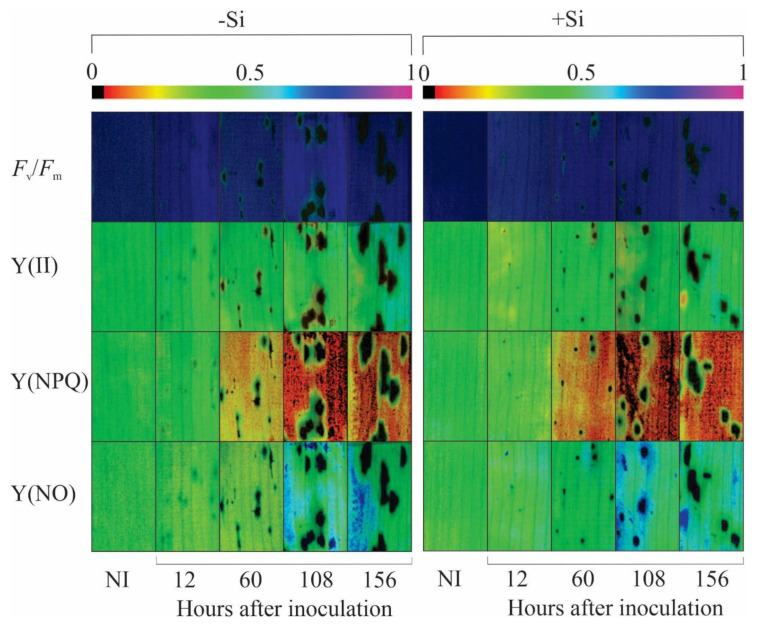
Images of chlorophyll *a* fluorescence parameters variable-to-maximum chlorophyll *a* fluorescence ratio (*F*_v_/*F*_m_), photochemical yield [Y(II)], yield for dissipation by downregulation [Y(NPQ)], and yield for non-regulated dissipation [Y(NO)] obtained from the leaves of maize plants non-supplied (−Si) or supplied (+Si) with silicon (Si) and non-inoculated (NI) or at different times after inoculation with *Bipolaris maydis* (isolate UFV-DPF-*Bm*12).

**Figure 6:**
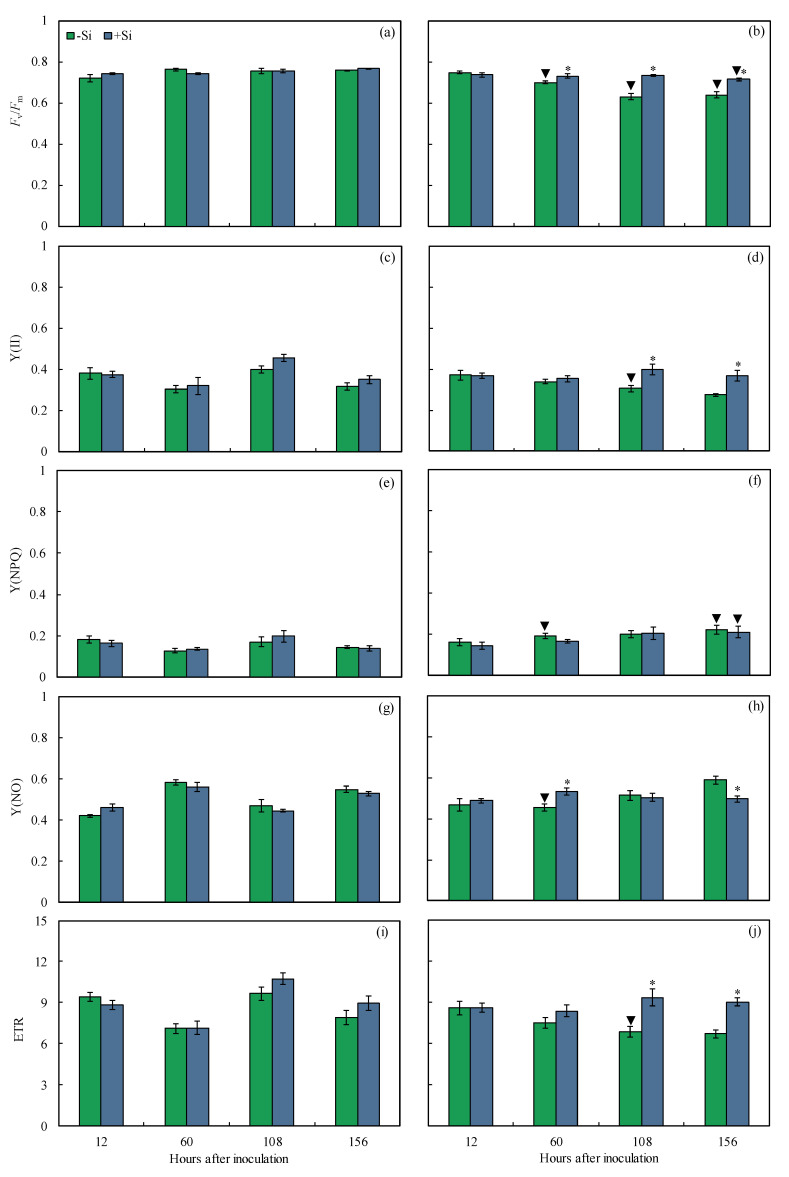
Quantification of chlorophyll *a* fluorescence parameters variable-to-maximum chlorophyll *a* fluorescence ratio (*F*_v_/*F*_m_) (**a**,**b**), photochemical yield [Y(II)] (**c**,**d**), yield for dissipation by downregulation [Y(NPQ)] (**e**,**f**), yield for non-regulated dissipation [Y(NO)] (**g**,**h**), and electron transport rate (ETR) (**i**,**j**) determined on the leaves of maize plants non-inoculated (NI) (**a**,**c**,**e**,**g**,**i**) or inoculated (I) (**b**,**d**,**f**,**h**,**j**) with *Bipolaris maydis* (isolate UFV-DPF-*Bm*12) and non-supplied (−Si) or supplied (+Si) with silicon (Si). Means for NI and I plants followed by an inverted triangle (▼) and for −Si and +Si treatments followed by an asterisk (*), at each evaluation time, are significantly different (*p* ≤ 0.05) according to the *F* test. Bars represent the standard error of the means.

In combination with the leaf gas exchange parameters, the Chl *a* fluorescence parameters will bring more insight into the functionality of the photosynthetic apparatus of +Si-infected plants in comparison to −Si infected plants. For inoculated plants, remarkable changes in the images for *F*_v_/*F*_m_, Y(II), Y(NPQ), and Y(NO), based on the darker areas, were noticed in the leaves of −Si plants compared to the leaves of +Si plants from 12 to 156 hai indicating damage to the photosynthetic apparatus (Figure 5). For inoculated +Si plants, *F*_v_/*F*_m_ (6, 14, and 10% at 60, 108, and 156 hai, respectively), Y(II) (24 and 26% at 108 and 156 hai, respectively), Y(NO) (15% at 60 hai), and ETR (27 and 26% at 108 and 156 hai, respectively) were significantly higher while Y(NO) (15% at 156 hai) was significantly lower compared to inoculated −Si plants (Figure 6b,d,f,h,j). For −Si plants, *F*_v_/*F*_m_ (8–16% from 60 to 156 hai), Y(II) (24% at 108 hai), Y(NO) (22% at 60 hai), and ETR (29% at 108 hai) were significantly lower while Y(NPQ) (33 and 35% at 60 and 156 hai, respectively) was significantly higher for inoculated plants compared to non-inoculated plants (Figure 6). For +Si plants, *F*_v_/*F*_m_ was significantly lower by 8% and Y(NPQ) was significantly higher by 34% at 156 hai for inoculated plants compared to non-inoculated plants (Figure 6a,b,e,f).

### 2.6. Photosynthetic Pigments

**Figure 7:**
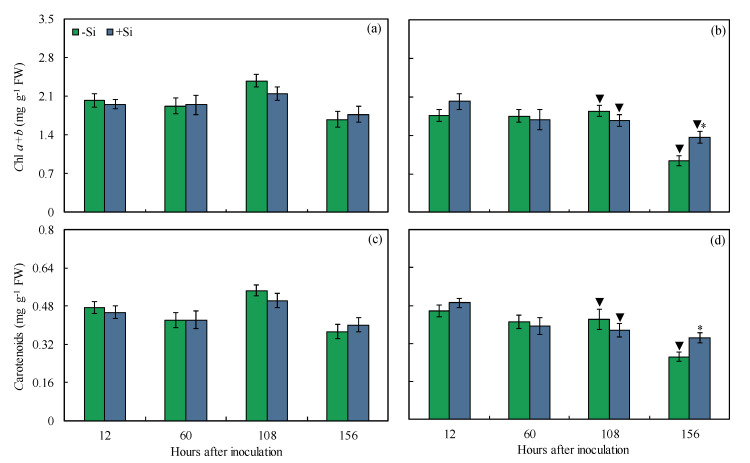
Concentrations of chlorophyll *a*+*b* (Chl *a+b*) (**a**,**b**) and carotenoids (**c**,**d**) determined on the leaves of maize plants non-inoculated (NI) (**a**,**c**) or inoculated (I) (**b**,**d**) with *Bipolaris maydis* (isolate UFV-DPF-*Bm*12) and non-supplied (−Si) or supplied (+Si) with silicon (Si). Means for NI and I plants followed by an inverted triangle (▼) and for −Si and +Si treatments followed by an asterisk (*), at each evaluation time, are significantly different (*p* ≤ 0.05) according to the *F* test. Bars represent the standard error of the means. FW = fresh weight.

The pool of photosynthetic pigments was determined in the leaves of non-inoculated and inoculated plants that were non-supplied or supplied with Si from 12 to 156 hai aiming to confirm the changes in both leaf gas exchange and Chl *a* fluorescence parameters. For inoculated +Si plants, the concentrations of Chl *a*+*b* and carotenoids (34 and 47% at 156 hai, respectively) were significantly higher compared to inoculated −Si plants (Figure 7b,d). For −Si plants, concentrations of Chl *a*+*b* (23 and 44% at 108 and 156 hai, respectively) and carotenoids (23 and 29% at 108 and 156 hai, respectively) were significantly lower for inoculated plants compared to non-inoculated plants (Figure 7a–d). For +Si plants, concentrations of Chl *a*+*b* (22 and 23% at 108 and 156 hai, respectively) and carotenoids (25 and 15% at 108 and 156 hai, respectively) were significantly lower for inoculated plants compared to non-inoculated plants (Figure 7a–d).

### 2.7. Carbohydrates

**Figure 8:**
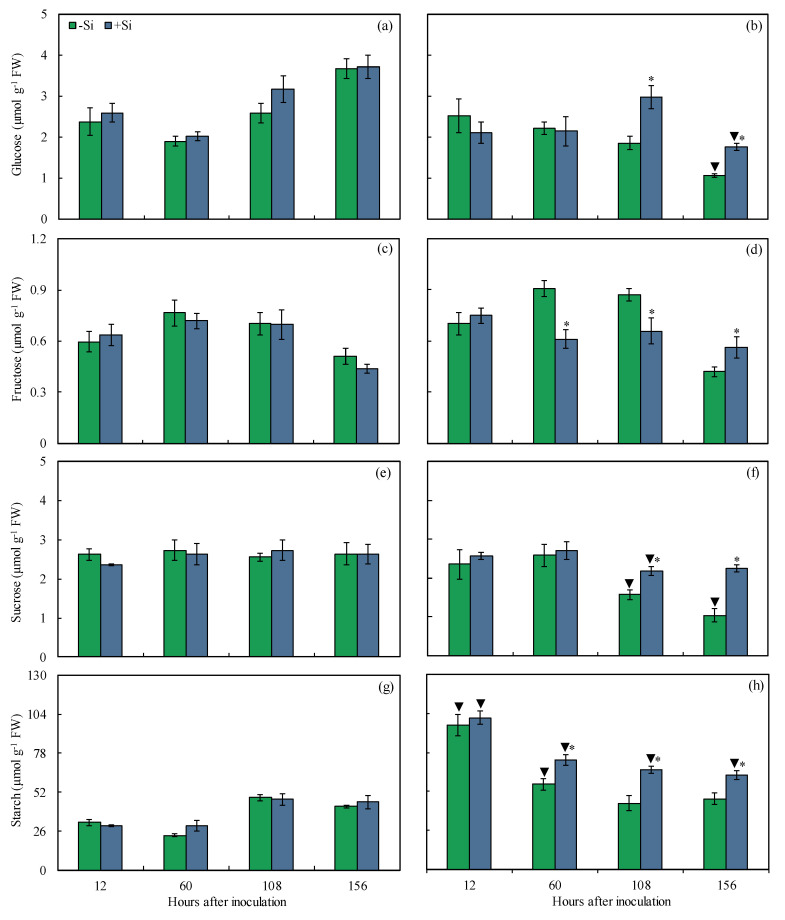
Concentrations of glucose (**a**,**b**), fructose (**c**,**d**), sucrose (**e**,**f**), and starch (**g**,**h**) determined on the leaves of maize plants non-inoculated (NI) (**a**,**c**,**e**,**g**) or inoculated (I) (**b**,**d**,**f**,**h**) with *Bipolaris maydis* (isolate UFV-DPF-*Bm*12) and non-supplied (−Si) or supplied (+Si) with silicon (Si). Means for NI and I plants followed by an inverted triangle (▼) and for −Si and +Si treatments followed by an asterisk (*), at each evaluation time, are significantly different (*p* ≤ 0.05) according to the *F* test. Bars represent the standard error of the means. FW = fresh weight.

The metabolism of sugars and starch was investigated in the leaves of −Si and +Si plants that were non-inoculated or inoculated with *B. maydis* (isolate UFV-DPF-*Bm*12) in a time-course experiment. This biochemical analysis allowed us to link the changes in the pool of these metabolites with the capacity of +Si plants to respond more efficiently against *B. maydis* infection. For inoculated +Si plants, glucose (38 and 39% at 108 and 156 hai, respectively), fructose (125% at 156 hai), sucrose (27 and 54% at 108 and 156 hai, respectively), and starch (23–34% from 60 to 156 hai) concentrations were significantly higher, while fructose concentration (33 and 24% at 60 and 108 hai, respectively) was significantly lower compared to inoculated −Si plants (Figure 8b,d,f,h). In −Si plants, concentrations of glucose (70% at 156 hai) and sucrose (38 and 60% at 108 and 156 hai, respectively) were significantly lower while starch concentration (67 and 58% at 12 and 60 hai, respectively) was significantly higher for inoculated plants compared to non-inoculated plants (Figure 8a,b,e–h). For +Si plants, concentrations of glucose (53% at 156 hai) and sucrose (20% at 108 hai) were significantly lower, while the concentration of starch (79, 59, 29, and 28% at 12, 60, 108, and 156 hai, respectively) was significantly higher for inoculated plants compared to non-inoculated plants (Figure 8a,b,e–h).

### 2.8. Concentrations of MDA, H_2_O_2_, and O_2_^•−^

**Figure 9:**
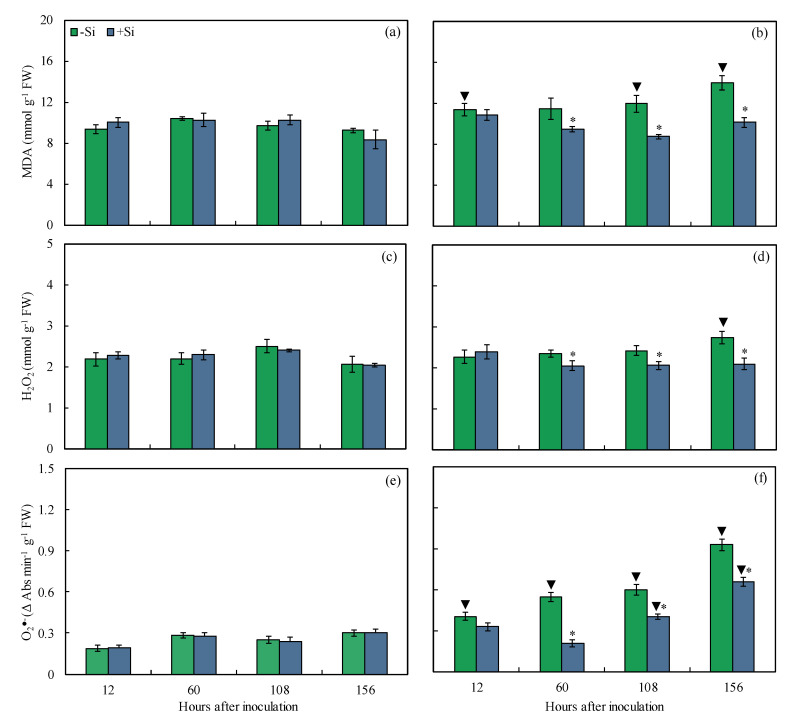
Concentrations of malondialdehyde (MDA) (**a**,**b**), hydrogen peroxide (H_2_O_2_) (**c**,**d**), and superoxide anion radical (O_2_^•−^) (**e**,**f**) determined on the leaves of maize plants non-inoculated (NI) (**a**,**c**,**e**) or inoculated (I) (**b**,**d**,**f**) with *Bipolaris maydis* (isolate UFV-DPF-*Bm*12) and non-supplied (−Si) or supplied (+Si) with silicon (Si). Means for NI and I plants followed by an inverted triangle (▼) and for −Si and +Si treatments followed by an asterisk (*), at each evaluation time, are significantly different (*p* ≤ 0.05) according to the *F* test. Bars represent the standard error of the means. FW = fresh weight.

The oxidative stress imposed on maize leaves in response to infection by *B. maydis* (isolate UFV-DPF-*Bm*12) was determined by measuring the pool of MDA, H_2_O_2_, and O_2_^•−^. The foliar levels of these metabolites were expected to be lower for +Si-infected plants in comparison to −Si infected plants in a time-course experiment. This biochemical analysis allowed us to link the changes in the pool of these metabolites with the capacity of +Si plants to respond more efficiently against *B. maydis* infection. For inoculated plants, MDA (18, 27, and 28% at 60, 108, and 156 hai, respectively), H_2_O_2_ (15, 15, and 24% at 60, 108, and 156 hai, respectively), and O_2_^•−^ (62, 33, and 29% at 60, 108, and 156 hai, respectively) concentrations were significantly lower for +Si plants compared to inoculated −Si plants (Figure 9b,d,f). For −Si plants, MDA (8, 44, and 34% at 12, 108, and 156 hai, respectively), H_2_O_2_ (25% at 156 hai), and O_2_^•−^ (47–68% from 12 to 156 hai) were significantly higher for inoculated plants compared to non-inoculated plants (Figure 9). For +Si plants, O_2_^•−^ was significantly higher by 40 and 53% at 108 and 156 hai, respectively, for inoculated plants compared to non-inoculated plants (Figure 9e,f).

### 2.9. Activities of Defense Enzymes

**Figure 10:**
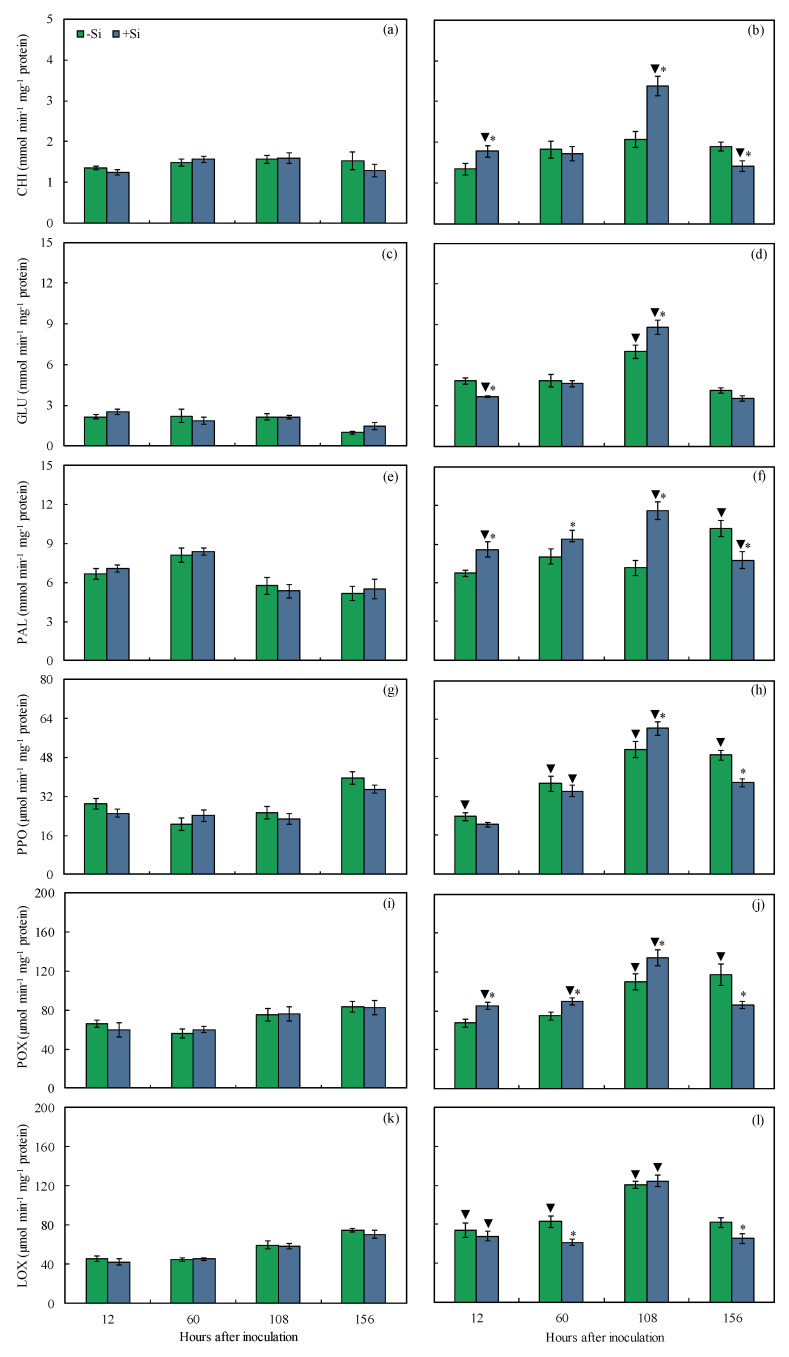
Activities of chitinase (CHI) (**a**,**b**), *β*-1,3-glucanase (**c**,**d**), phenylalanine ammonia-lyase (PAL) (**e**,**f**), polyphenoloxidase (PPO) (**g**,**h**), peroxidase (POX) (**i**,**j**), and lipoxygenase (LOX) (**k**,**l**) determined on the leaves of maize plants non-inoculated (NI) (**a**,**c**,**e**,**g**) or inoculated (I) (**b**,**d**,**f**,**h**) with *Bipolaris maydis* (isolate UFV-DPF-*Bm*12) and non-supplied (−Si) or supplied (+Si) with silicon (Si). Means for NI and I plants followed by an inverted triangle (▼) and for −Si and +Si treatments followed by an asterisk (*), at each evaluation time, are significantly different (*p* ≤ 0.05) according to the *F* test. Bars represent the standard error of the means.

The direct or indirect response of maize leaf cells to infection by *B. maydis* (isolate UFV-DPF-*Bm*12) was attested by measuring the activities of defense enzymes^−^. The foliar activities of these enzymes were determined in the leaves of −Si and +Si plants that were non-inoculated or inoculated with *B. maydis* (isolate UFV-DPF-*Bm*12) in a time-course experiment. The CHI (24 and 38% at 12 and 108 hai, respectively), GLU (20% at 108 hai), PAL (15–38% from 12 to 108 hai), PPO (15% at 108 hai), and POX (17–21% from 12 to 108 hai) activities were significantly higher for inoculated +Si plants compared to inoculated −Si plants (Figure 10b,d,f–h,j). The CHI, PAL, PPO, and POX activities were significantly lower at 156 hai for inoculated +Si plants than inoculated −Si plants (Figure 10b,f,h,j). The GLU (24% at 12 hai) and LOX (26 and 20% at 60 and 156 hai, respectively) activities were significantly lower for inoculated +Si plants compared to inoculated −Si plants (Figure 10d,l). The GLU (69% at 108 hai), PAL (49% at 156 hai), PPO (44, 51, and 20% at 60, 108, and 156 hai, respectively), POX (31 and 28% at 108 and 156 hai, respectively), and LOX (38, 46, and 51% at 12, 60, and 108 hai, respectively) activities were significantly higher for inoculated −Si plants compared to non-inoculated −Si plants (Figure 10c–l). The activities of CHI (31 and 25% at 12 and 108 hai, respectively), GLU (76% at 108 hai), PAL (17 and 54% at 12 and 108 hai, respectively), PPO (29 and 62% at 60 and 108 hai, respectively), POX (30, 33, and 43% at 12, 60, and 108 hai, respectively), and LOX (37 and 53% at 12 and 108 hai, respectively) were significantly higher for inoculated +Si plants compared to non-inoculated +Si plants (Figure 10a–l).

### 2.10. Concentrations of TSP and LTGA Derivatives

**Figure 11:**
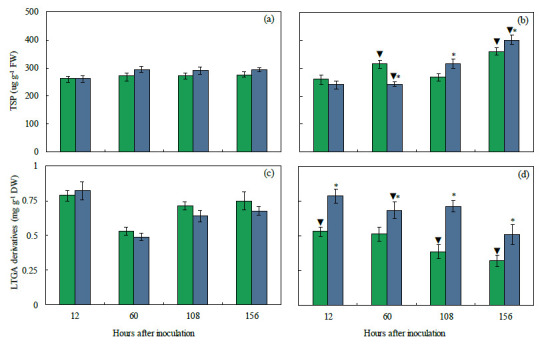
Concentrations of total soluble phenolics (TSP) (**a**,**b**) and lignin-thioglycolic acid (LTGA) derivatives (**c**,**d**) determined on leaves of the maize plants non-inoculated (NI) (**a**,**c**) or inoculated (I) (**b**,**d**) with *Bipolaris maydis* (isolate UFV-DPF-*Bm*12) and non-supplied (−Si) or supplied (+Si) with silicon (Si). Means for NI and I plants followed by an inverted triangle (▼) and for −Si and +Si treatments followed by an asterisk (*), at each evaluation time, are significantly different (*p* ≤ 0.05) according to the *F* test. Bars represent the standard error of the means. FW and DW = fresh weight and dry weight, respectively.

**Figure 12:**
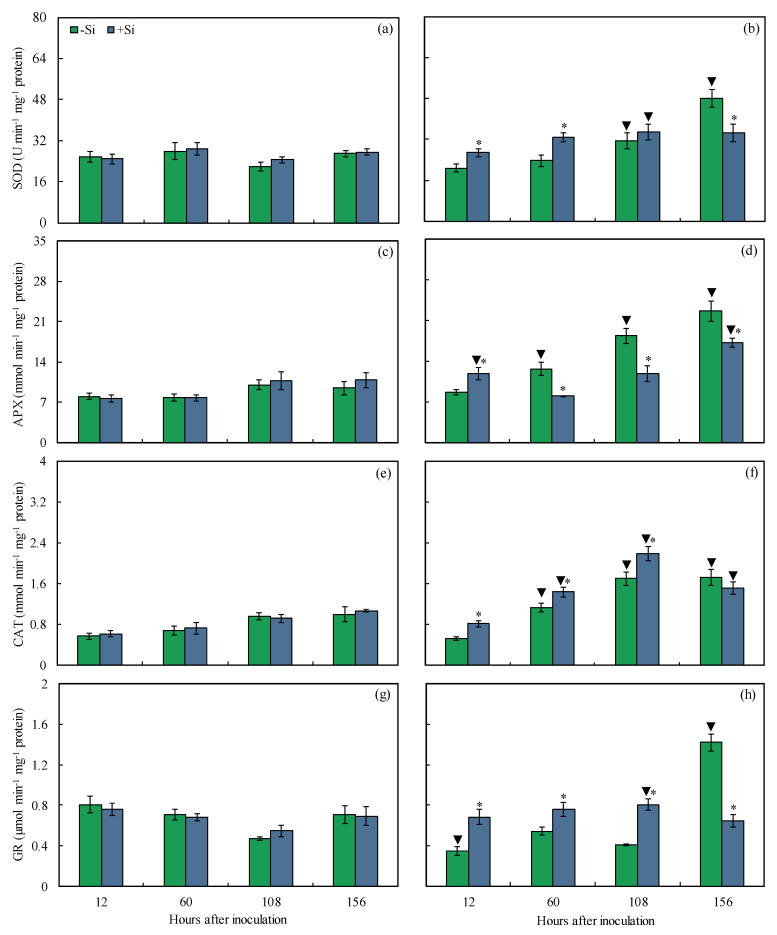
Activities of superoxide dismutase (SOD) (**a**,**b**), ascorbate peroxidase (APX) (**c**,**d**), catalase (CAT) (**e**,**f**), and glutathione reductase (GR) (**g**,**h**) determined on the leaves of maize plants non-inoculated (NI) (**a**,**c**,**e**,**g**) or inoculated (I) (**b**,**d**,**f**,**h**) with *Bipolaris maydis* (isolate UFV-DPF-*Bm*12) and non-supplied (−Si) or supplied (+Si) with silicon (Si). Means for NI and I plants followed by an inverted triangle (▼) and for −Si and +Si treatments followed by an asterisk (*), at each evaluation time, are significantly different (*p* ≤ 0.05) according to the *F* test. Bars represent the standard error of the means.

The foliar concentrations of phenolics and lignin (indicated by the pool of LTGA derivatives), linked to PAL activity, will dictate the level of response of +Si plants to infection by *B. maydis* (isolate UFV-DPF-*Bm*12). The foliar levels of these metabolites were determined in a time-course experiment using −Si and +Si plants that were non-inoculated and inoculated with *B. maydis*. For inoculated plants, the concentrations of TSP (16 and 11% at 108 and 156 hai) and LTGA derivatives were significantly higher (33, 25, 33, and 37% at 12, 60, 108, and 156 hai, respectively) for inoculated +Si plants compared to inoculated −Si plants (Figure 11b,d). The TSP concentration was significantly higher (13 and 23% at 60 and 156 hai, respectively), while the LTGA derivatives concentration was significantly lower (33, 45, and 57% at 12, 108, and 156 hai, respectively) for inoculated −Si plants compared to non-inoculated −Si plants (Figure 12a–d). The concentrations of TSP (27% at 156 hai) and LTGA derivatives (28% at 60 hai) were significantly higher for inoculated +Si plants compared to non-inoculated +Si plants (Figure 11a–d).

### 2.11. Antioxidant Enzymes

A time-course experiment (from 12 to 156 hai) was carried out to evaluate the changes in activities of antioxidant enzymes in leaves of −Si and +Si plants that were non-inoculated or inoculated with *B. maydis* (isolate UFV-DPF-*Bm*12). For inoculated plants, SOD (22 and 28% at 12 and 60 hai, respectively), APX (26% at 12 hai), CAT (21–35% from 12 to 108 hai), and GR (28–49% from 12 to 108 hai) activities were significantly higher for +Si plants compared to −Si plants (Figure 12b,d,f,h). There were significant increases in SOD (28% at 156 hai), APX (24–37% from 60 to 156 hai), and GR (55% at 156 hai) activities for inoculated −Si plants compared to inoculated +Si plants (Figure 12b,d,h). Activities of SOD (30 and 44% at 108 and 156 hai, respectively), APX (38, 45, and 58% at 60, 108, and 156 hai, respectively), CAT (40, 44, and 42% at 60, 108, and 156 hai, respectively), and GR (50% at 156 hai) were significantly higher for inoculated −Si plants compared to non-inoculated −Si plants (Figure 12a–h). Activities of SOD (29% at 108 hai), APX (35 and 37% at 12 and 156 hai, respectively), CAT (48, 58, and 30% at 60, 108, and 156 hai, respectively), and GR (32% at 108 hai) were significantly higher for inoculated +Si plants compared to non-inoculated +Si plants (Figure 12a–h).

## 3. Discussion

It is well known that Si plays a crucial role in plant resistance against an array of pathogens infecting various profitable crops [12,13,14,17]. In this study, new evidence at the physiological and biochemical levels clearly indicated the role of Si in reducing MLB symptoms. Higher foliar Si concentration for +Si plants reduced the progress of MLB which is associated with lower concentrations of MDA, H_2_O_2_, and O_2_^●−^. In this scenario, leaf cells were protected from oxidative damage caused by fungal infection. Different reports showed that the intensity of many diseases in gramineous plants such as rice, sugarcane, wheat, and maize was negatively correlated with higher foliar Si concentration when this element was supplied to plants through the roots [12,13,19,20]. In maize, the uptake and translocation of Si from the roots to the shoots are governed by the presence of the transporters ZmLsi1 and ZmLsi6, which operate in the active Si uptake pathway independent of a passive mechanism that occurs through the transpiratory flux of water [16,17].

Photosynthesis is the physiological process most negatively affected in plants infected by pathogens of different lifestyles reducing the efficiency of light capture, energy production, and the synthesis of photoassimilates [21,22,23]. In the present study, Si supply reduced the negative effect of *B. maydis* infection on the photosynthetic capacity of maize plants, as indicated by the higher values of *A*, *g*_s_, and *E*. In contrast, Si had a significant effect on *C*_i_ only at 108 hai for infected leaves. In general, +Si-infected plants showed a proportional increase in *A* and *g*_s_ simultaneously with unchanged values for *C*_i_ at 156 hai indicating that increases in *A* values during the infection process of *B. maydis* in the leaves of +Si plants were not strongly associated with limitations in CO_2_ influx into the stomata for photosynthesis. Thus, changes in *A* were probably not caused by the obtained values for *g*_s_, considering the concomitant increases in the values of these parameters throughout the *B. maydis* infection process without strong changes in *C*_i_. Therefore, the effect of Si to mitigate the decreases in *A* should be explained by alleviating the dysfunctions that may take place at the level of biochemical reactions in the chloroplasts that involve CO_2_ fixation for a better photosynthetic performance of maize plants. Similar reports have shown the positive effect of Si on keeping a better photosynthetic performance by monitoring the changes in the leaf gas exchange parameters in the interactions of rice-*Pyricularia oryzae* and rice-*Monographella albescens* [24,25], as well as wheat-*P. oryzae* [26]. The impairment of *A* in maize plants may also have been associated with the release of hydrolytic enzymes and non-host selective toxins by *B. maydis* into the leaf tissues that can lower CO_2_ assimilation due to the destruction of stomata, damage to the photosynthetic apparatus, and degradation of photosynthetic pigments [27]. However, these dysfunctions were attenuated by Si due to the formation of a thick silica layer below the cuticle that acted as a physical barrier [12,13] that inhibited or delayed *B. maydis* penetration and its growth over the leaf surface, as shown by the X-ray microanalysis, contributing, therefore, to the preservation of the photosynthetic apparatus.

In addition to the impact on the biochemical function of CO_2_ fixation, changes in the photochemical capacity of photosynthesis were more prominent at later stages of *B. maydis* infection for −Si plants. Based on the imaging of Chl *a* fluorescence parameter, early damage of fungal infection against photosynthesis can be visualized non-invasively before the symptoms are noticed [22]. The parameters *F*_v_/*F*_m_ and Y(II) represent the capacity of photon energy absorbed by the PSII to be used in photochemical processes and are considered physiological markers to understand the effect of biotic stress-induced photoinhibition and the level of disease impact on photosynthesis [28,29]. In addition, photoinhibition occurring at the PSII level may affect the redox balance of electron transport components to produce reducing power and consequently the deactivation of RuBisCO [30]. Interestingly, the infected leaves of +Si plants suffered less photooxidative damage as clearly indicated by the higher values of *F*_v_/*F*_m_, Y(II), and ETR concomitantly without significant changes in Y(NPQ) and Y(NO) values, indicating, therefore, that the functionality of the photosynthetic apparatus was preserved. The higher linear electron flow and the Y(II) parameter indicate a key role of the dissipative mechanisms mainly modulated by Si to bring sufficient photochemical energy to be used to support the higher photosynthetic performance of leaves infected with *B. maydis*. The maize and wheat plants supplied with Si and infected by *E. turcicum* and *P. oryzae,* respectively [20,24], showed higher values of *F*_v_/*F*_m_, Y(II), and ETR, indicating the positive contribution of this element to reduce the dysfunctions at the photochemical level. Plants under stress and supplied with Si displayed a greater amount of open PSII reaction centers that resulted in higher excitation energy in the electron flow, thus improving the photochemical efficiency of photosynthesis [31]. Studies at the transcriptomic level in *Brassica napus* [32] and *Cucumis sativus* [33] showed that Si promoted the differential expression of genes encoding proteins that were involved in photochemical reactions, mainly those associated with the light-harvesting complex, PSII, and the electron transport chain resulting in better integrity of chloroplasts and the photosynthetic apparatus overall.

In addition to the parameters related to Chl *a* fluorescence, the quantification of the pool of photosynthetic pigments is a stronger indicator of the decreased efficiency of the photosynthetic apparatus of plants infected by hemibiotrophic and necrotrophic pathogens due to the action of hydrolytic enzymes and selective non-host toxins that directly affect chloroplasts and their protein complexes [28,34]. The loss of functionality of the photosynthetic pigments is one of the main effects caused by *B. maydis* infection in maize leaves [8]. The reduction of MLB symptoms in the leaves of +Si plants was closely linked to a higher pool of photosynthetic pigments (Chl *a*+*b* and carotenoids) at 156 hai, a later stage on *B. maydis* infection process, evidencing less damage to the photosynthetic apparatus. Previous studies emphasized that Si played a crucial role in maintaining the integrity of chloroplasts in the leaves of plants exposed to cellular oxidative stress caused by pathogens infection, which resulted in the preservation of the pool of photosynthetic pigments [17,18].

Besides photosynthesis, the metabolism of carbohydrates in plants plays a detrimental role during the pathogen infection process [35]. The metabolism of soluble sugar represents a very complex physiological process in several host–pathogen interactions considering that the flow of photoassimilates to trigger defense reactions at the pathogen infection sites is extensively demanded by the plants [28,35]. In addition, changes in the levels of photoassimilates may greatly affect the pathogen infection process, considering that sugars serve as nutrients and signals during the colonization of host tissues by the pathogens [36]. In the present study, the downregulation of photosynthesis may have reduced the pool of sugars and starch in the infected leaves of −Si plants that displayed expressive symptoms of MLB. In general, for infected +Si plants, higher concentrations of glucose and sucrose were found at later stages of fungus infection while starch concentration was kept higher almost during the entire time-course evaluated. These findings clearly indicate a possible effect of Si in enhancing maize resistance against MLB through the modulation of carbohydrate metabolism rather than favoring fungal infection. In rice, Si conferred resistance to brown spots by regulating the expression of photosynthetic genes (e.g., RuBisCO production) and those related to nitrate and nitrite reductases, as well as nitrate transporter [37]. Rice plants supplied with Si showed increased sucrose concentration in response to *B. oryzae* infection [38]. The expression of Calvin cycle-related genes such as those coding for fructose-1,6-bisphosphate aldolase, phosphate isomerase, sedoheptulose-1,7-bisphosphatase, glyceraldehyde-3-phosphate dehydrogenase, fructose-1,6-bisphosphatase, and RuBisCO was repressed in cucumber plants infected with *Fusarium oxysporum* [39]. However, the expression of the genes mentioned above increased and improved the pool of soluble sugars and the resistance of cucumber against Fusarium wilt for Si-supplied plants. Interestingly, fructose concentration for infected +Si plants decreased at earlier times of *B. maydis* infection and increased later. Similar results for fructose accumulation were found for wheat plants supplied with Si and infected with *P. oryzae* indicating a possible use of fructose to favor fungal infection [40]. Considering these findings related to the regulatory role of Si on physiological attributes conferring increased resistance of maize against MLB, the pattern of both sugar metabolism and photosynthetic capacity can be used as benchmark markers for the positive effect of Si on maize-*B. maydis* interaction.

It is well known that the accumulation of MDA in host tissues infected by pathogens results from an intense lipid peroxidation on cell membrane caused by an excess of H_2_O_2_ or O_2_^●−^ [41]. In response to this oxidative stress due to pathogen infection, an enzymatic antioxidant system with the action of SOD, APX, CAT, and GR activities takes place to keep the generation of reactive oxygen species (ROS) at physiologically accepted levels [42]. In the present study, MDA concentration was lower for infected leaves of +Si plants from 60 to 156 hai and was associated with reduced production of H_2_O_2_ or O_2_^●−^. In addition, SOD, APX, CAT, and GR activities were greatest earlier in the *B. maydis* infection process while SOD, CAT, and GR activities were higher at the advanced stage of fungal infection. Other studies showed that Si can stimulate the enzymatic antioxidant metabolism of pathogen-challenged plants by restricting the oxidative stress caused by an excess of ROS associated with less lipid peroxidation in the infected cells [12,13,17,20,39,43]. In the present study, APX activity was higher for infected −Si plants in contrast to infected +Si plants from 60 to 156 hai, possibly due to the higher availability of H_2_O_2_ that needed detoxification. The greater concentration of H_2_O_2_ accompanied by O_2_^●−^ may indirectly indicate oxidative stress (e.g., action of several hydrolytic enzymes and non-host selective toxins) generated by the colonization of maize leaves of −Si plants by *B. maydis*. Both CAT and APX act in the elimination of H_2_O_2_, however, it has been suggested that moderate concentrations of H_2_O_2_ are eliminated mainly by APX with the aid of various reductants such as ascorbate and glutathione [44].

Biochemically, the enhancement of the resistance of crops challenged by pathogens by Si is associated with the joint action of defense-related enzymes and the production of phenolic compounds [12,13,17,45]. The defense proteins CHI and GLU, which catalyze the hydrolysis of key fungal cell wall components (chitin and β-1,3-glucan, respectively), are important for the resistance of several plant species infected by fungal pathogens of different lifestyles [46]. In the present study, CHI activity was highest at 12 hai while GLU was highest only at 108 hai for infected +Si plants. The results of the present study are corroborated by the findings of Fortunato et al. [47], who found higher CHI and GLU activities in the roots of banana plants supplied with Si. Products of secondary metabolism such as phenolic compounds, lignin, phytoalexins, and flavonoids are dependent on the initial activity of PAL through the phenylpropanoid pathway [48]. The PPO and POX play key roles in the oxidation of polyphenols to quinones, which are highly toxic against several, and in the lignification of cell walls with a cautious participation of H_2_O_2_ [49]. In the present study, PAL and POX activities followed the same increasing trend from 12 to 108 hai, but PPO activity was higher only at 108 hai for infected +Si plants. The Si-mediated resistance in perennial ryegrass against *Magnaporthe oryzae* infection was associated with higher activities of PAL, POX, and PPO [50]. Increased LOX activity is known to occur in response to the oxidation of fatty acids released by ROS-induced lipid peroxidation that leads to the biosynthesis of jasmonic acid, considered a signaling molecule for induced systemic resistance against pathogen attack [51]. In the present study, lower LOX activity was evidenced for infected −Si plants at 60 and 156 hai. The low LOX activity may be related to lower levels of lipid peroxidation and ROS accumulation during the infection of leaves from +Si plants by *B. maydis* [52].

For +Si plants, the production of LTGA derivatives increased throughout the *B. maydis* infection process, while higher TSP concentration occurred only at the later stages of fungal infection. It is known that plants supplied with Si display an increased concentration of phenolics and lignin due to a potentiation of the phenylpropanoid pathway to efficiently counteract the infection process of pathogens of different lifestyles [12,13,14,17,53]. For example, in maize leaves and banana roots infected with *S. macrospora* and *F. oxysporum* f. sp. *cubense*, respectively, Si potentiated the phenylpropanoid pathway by increasing the production of phenolic compounds and lignin and significantly reducing the damage caused by these pathogens on host tissues [19,47]. The PAL and POX are pivotal enzymes for the biosynthesis of phenolic compounds and lignin [48,49] and their higher activities, as evidenced in the present study may have greatly contributed to the strengthening of the cell wall in the leaves of +Si plants that may impair the colonization process of *B. maydis*. On top of that, greater production of carbohydrates may have also contributed as respiratory substrates to be used in the formation of energy-rich compounds (e.g., ATP), as well as intermediate compounds that could be linked to the increased resistance of leaves of +Si plants to MLB.

Taken together, the results of the present study integrate physiological and biochemical pieces of evidence for the beneficial role played by Si in enhancing the resistance of leaves of maize against MLB. In this regard, plants supplied with Si had a more preserved photosynthetic apparatus, a more robust antioxidative metabolism, greater response of defense-related enzymes, and regulation of carbohydrate metabolism that worked all together in favor of the increased resistance of leaves from +Si plants infected by *B. maydis*.

## 4. Material and methods

### 4.1. Plant Material, Fertilizantion, and Growth Conditions

Seeds of the maize hybrid B2433PWU (Brevant, São Paulo, Brazil), susceptible to *B. maydis*, were sown in plastic pots containing 2 kg of substrate (1:1:1 mixture of pine bark, peat, and expanded vermiculite; Tropstrato^®^, Vida Verde, São Paulo, Brazil). The concentration of Si in the substrate was 4.6 mg kg^−1^. A total of 1.63 g of calcium phosphate was added to each pot to provide phosphorus to plants before sowing. Only two plants were kept per pot after the emergence of seedlings (≈five days). Plants were fertilized with the nutrient solution (100 mL per pot twice a week) proposed by Hoagland and Arnon [40], with a few modifications, as follows: 2.6 mM KCl, 0.6 mM K_2_SO_4_, 1.2 mM MgSO_4_, 1.0 mM CH_4_N_2_O, 1.2 mM NH_4_NO_3_, 0.0002 mM (NH_4_)_6_Mo_7_O_24_, 0.03 mM H_3_BO_4_, 0.04 mM ZnSO_4_, 0.01 mM CuSO_4_, 0.03 mM MnCl_2_, 0.015 mM FeSO_4_, and 0.015 mM ethylenediaminetetraacetic acid disodium (EDTA). Potassium silicate (PS) (FertiSil^®^, PQ Corporation, São Paulo, Brazil; 13% K_2_O and 26.59% SiO_2_) was used as the Si source. The two plants per pot received 200 mL of a PS solution (0.34 mL PS/L; 2 mM Si, pH 6.5 ± 0.2 by adding either an HCl or NaOH solution at 0.5 M) daily for 30 days. For the control treatment, plants in each pot received 200 mL of a KCl solution (1.19 mL KCl/L; 1.53 mM K, pH 6.5 ± 0.2 adjusted as mentioned above) daily for 30 days. The K concentration between plants receiving either PS or KCl was kept the same (1.53 mM K). All solutions were prepared using deionized water. Plants were kept in the greenhouse (temperature of 28 ± 5 °C, relative humidity of 80 ± 5%, and natural photosynthetically active radiation (PAR) of 900 ± 15 μmol photons m^−2^ s^−1^ measured at midday).

### 4.2. Inoculum Production and Plant Inoculation

Stripes of filter paper containing fungal mycelia of the monosporic isolate of *B. maydis* UFV-DPF-*Bm*12 were transferred to Petri dishes containing potato-dextrose-agar. The dishes were incubated in a growth chamber (25 °C and 12 h light/12 h dark photoperiod) for 15 days. Conidia produced in each dish were collected using sterile water containing 0.01% Tween 20 and 0.5% gelatin (*w*/*v*). Using a hemacytometer, the conidial suspension was calibrated to 1 × 10^3^ conidia/mL. Plants at the V6 growth stage (30 days after emergence) were inoculated with a conidial suspension of *B. maydis* using a VL Airbrush atomizer (Paasche Airbrush Co., Chicago, IL, USA). After inoculation, plants were kept inside a mist growth chamber (25 °C and relative humidity of 90 ± 5%) for 24 h. After this period, plants were transferred to the greenhouse (28 ± 2 °C, relative humidity of 80 ± 5%, and natural PAR of 900 ± 15 μmol photons m^−2^ s^−1^ measured at midday) until the end of the experiments.

### 4.3. Evaluation of MLB Severity

The fifth expanded leaf, from base to top, of each plant per replication of each treatment was collected at 156 h after inoculation (hai), scanned at 600 dpi resolution, and the images were processed using the QUANT software [54] to estimate the values based on the development of disease symptoms (number and size of necrotic lesions, lesions coalescence, and chlorosis).

### 4.4. Determination of Foliar Si and K Concentrations and Their Detection Using X-ray Microanalysis

At the end of the experiment (156 hai), the fifth and sixth leaves, from base to top, of each plant per replication of each treatment were collected, washed in deionized water, dried for 72 h at 65 °C, and ground in a ball mill (TECNAL TE 350, Piracicaba, SP, Brazil) for 2 min. The Si concentration was determined by the colorimetry method with hydrochloric acid, oxalic acid, and ammonium molybdate in a spectrophotometer at 410 nm [55]. The K concentration was determined by digestion in nitric perchloric acid solution followed by an optical emission spectrophotometer with inductively coupled plasma reading (ICP-OES; DV8300) (PerkinElmer, Shelton, CT, USA) [56].

The energy dispersive X-ray spectroscopy (EDS) using a scanning electron microscope (LEO 1430VP; Carl Zeiss, Oberkochen, Baden-Württemberg, Germany) with an attached X-ray detector system (Tracor TN5502, Middleton, WI, USA) was used to determine the insoluble composition and relative levels of Si and K in the adaxial surface of infected leaves from −Si and +Si plants at 156 hai. Leaf samples were carefully placed in envelopes inside a desiccator with silica gel for 15 days. After complete dehydration, a total of four leaf fragments (≈1 × 1 cm^2^) containing MLS lesions were mounted onto one aluminum stub (two stubs per treatment) and coated with a thin film of evaporated carbon (Quorum Q150 T, Laughton, England). The EDS microanalysis of all samples was performed at the magnification of 200 × with an accelerating voltage of 20 kV and a working distance of 10 mm. The intensities and distribution patterns of Si and K were based on secondary electron images, X-ray emission spectra, and corresponding X-ray elemental maps compared to other chemical elements such as carbon, oxygen, magnesium, phosphorus, sulfur, and chlorine [57,58]. A total of six images were obtained from the fragments of each treatment.

### 4.5. Determination of Leaf Gas Exchange Parameters

The measurement of the net carbon assimilation rate (*A*), stomatal conductance to water vapor (*g*_s_), internal CO_2_ concentration (*C*_i_), and transpiration rate (*E*) parameters provide insights into how efficiently plants are able to carry out photosynthesis. *A* is the rate at which the carbon dioxide (CO_2_) is assimilated by leaves to produce oxygen in the presence of light and directly reflects the efficiency of photosynthesis; *g*_s_ measures how easily the CO_2_ can move through the stomata and influences its rate of uptake, water loss through transpiration, and the balance between water and carbon assimilation; *C*_i_ indicates the amount of CO_2_ inside the leaf tissues and reflects the efficiency of the internal processes involved in photosynthesis; and *E* indicates the amount of water that is evaporated from the leaf surface [59]. These parameters were measured on the fifth leaf, from base to top, of each plant per replication of each treatment at 12, 60, 108, and 156 hai from 09:00 to 12:00 h using a portable open-system infrared gas analyzer (LI-6400, LI-COR Inc., Lincoln, NE, USA). These parameters were also evaluated on the same leaves of non-inoculated plants at the evaluation times mentioned above. All measurements were carried out under the following conditions: leaf temperature controlled at 25 °C, chamber CO_2_ concentration of 420 ppm, PAR of 1200 μmol m^−2^ s^−1^, and the amount of blue light set with 10% of PAR to optimize stomatal aperture [60].

### 4.6. Imaging and Quantification of Chl a Fluorescence Parameters

The Imaging-PAM fluorometer and the Imaging Win software MAXI version (Heinz Walz GmbH, Eichenring, Germany) were used to obtain the images and parameters of Chl *a* fluorescence using the fifth leaf, from base to top, of each plant per replication of each treatment at 12, 60, 108, and 156 hai and also from non-inoculated plants at these same evaluation times. The methodology described by Fagundes-Nacarath et al. [54] was used to determine the parameters variable-to-maximum chlorophyll *a* fluorescence ratio (*F*_v_/*F*_m_), photochemical yield [Y(II)], yield for dissipation by downregulation [Y(NPQ)], yield for non-regulated dissipation [Y(NO)], and electron transport rate (ETR).

### 4.7. Determination of Photosynthetic Pigments Concentration

The concentrations of Chl *a*, Chl *b*, and carotenoids were determined using methanol as the solvent. Leaf tissue (50 mg) obtained from the leaves used to determine the parameters of Chl *a* fluorescence was ground in liquid nitrogen using a vibration ball mill (Retsch, Haan, Germany), and the fine powder was homogenized in 700 μL of methanol. The supernatant was used to quantify concentrations of Chl *a*, Chl *b*, and carotenoids at 470, 653, and 666 nm, respectively, using a saturated methanol solution as a blank [61].

### 4.8. Biochemical Assays

The fifth leaf, from base to top, of each plant per replication of each treatment (−Si and +Si plants that were non-inoculated or inoculated with *B. maydis*) was collected at 12, 60, 108, and 156 hai. Leaves from non-inoculated plants were sampled at these same evaluation times. Leaf samples were kept in liquid nitrogen during sampling and stored at −80 °C for the following biochemical analysis: (*i*) determination of concentrations of sugars, starch, malondialdehyde, hydrogen peroxide (H_2_O_2_), superoxide anion radical (O_2_^•−^), total soluble phenolics (TSP), and lignin thioglycolic acid (LTGA) derivatives concentrations, and (*ii*) activities of defense and antioxidant enzymes. The results of these biochemical assays will allow to contrast the four above-mentioned treatments to determine whether Si can help decrease MLB severity on maize plants. The pool of sugars will indicate the efficiency of photosynthesis; starch content will indicate whether energy will be allocated and utilized for defense reactions; the MDA concentration will show the level of oxidative stress on leaf tissues; the concentrations of TSP and LTGA derivatives along with the activities of defense-related enzymes will reflect the level of defense of plant tissues; and the activities of antioxidative enzymes linked to the pool of H_2_O_2_ and O_2_^•−^ will display the capacity of leaf tissues to overcome the deleterious effect of reactive oxygen species.

#### 4.8.1. Determining Sugars and Starch Concentrations

Leaf tissue (50 mg) was ground into a fine powder as described above and mixed with 700 µL of methanol at 80 °C for 20 min. Sugars (glucose, fructose, and sucrose) were determined in the soluble phase of the methanolic solution, while starch was determined in the pellet [62].

#### 4.8.2. Determining MDA Concentration

Leaf tissue (100 mg) was ground as described above and homogenized in 2 mL of 0.1% (*w*/*v*) trichloroacetic acid solution and the homogenate was centrifuged at 12,000× *g* for 15 min at 4 °C. After centrifugation, 250 µL of the supernatant was added to 750 µL of thiobarbituric acid solution and homogenized in a thermomixer for 30 min at 95 °C. The samples were centrifuged at 9000× *g* for 10 min and the absorbances were obtained at 600 and 532 nm [63].

#### 4.8.3. Determining H_2_O_2_ and O_2_^•−^ Concentrations

Leaf tissue (100 mg) was ground as described above and homogenized in 1 mL of a mixture containing 50 mM potassium phosphate buffer (pH 6.5) and 1 mM hydroxylamine. The homogenate was centrifuged at 10,000× *g* for 15 min at 4 °C. The supernatant was used to determine H_2_O_2_ concentration following the procedures of Dias et al. [64]. Leaf tissue (100 mg) was ground as described above and the fine powder was homogenized in 1 mL of a solution containing 100 mM potassium phosphate buffer (pH 7.2) and 1 mM sodium diethyldithiocarbamate. The homogenate was centrifuged at 22,000× *g* for 20 min at 4 °C and the supernatant was used to determine O_2_^•−^ concentration [65].

#### 4.8.4. Determining Defense-Related Enzymes Activities

Leaf tissue (100 mg) was ground as described above and homogenized in 1 mL of a solution containing 50 mM potassium phosphate buffer (pH 6.8), 0.1 mM EDTA, 1 mM phenylmethylsulfonyl fluoride (PMSF), and 0.5% (*w*/*v*) polyvinylpyrrolidone (PVP). The homogenate was centrifuged at 13,000× *g* for 15 min at 4 °C and the supernatant was used to determine chitinase (CHI; EC 3.2.1.14), *β*-1,3-glucanase (GLU; EC 3.2.1.39), phenylalanine ammonia-lyase (PAL; EC 4.3.1.24), polyphenoloxidase (PPO; EC 1.10.3.1), peroxidase (POX; EC 1.11.1.7), and lipoxygenase (LOX; EC 1.13.11.12) activities following the procedures described by Fortunato et al. [47]. Briefly, CHI activity was assayed following the colorimetric determination of *p*-nitrophenyl cleaved from a chitin-analogous substrate p-nitrophenyl-β-D-N,N′-diacetylchitobiose. The GLU activity was determined using laminarin as the substrate in the reaction mixture to obtain reducing sugars that were determined by using dinitrosalicylic acid. For PAL activity, *L*-phenylalanine was used as the substrate in the reaction mixture and the absorbance of the trans-cinnamic acid derivatives was recorded at 290 nm. The POX activity was assayed following the colorimetric determination of pyrogallol oxidation in the reaction mixture based on the absorbance of the colored purpurogalin recorded at 420 nm. The PPO activity was determined in the same manner as for POX activity, except that H_2_O_2_ was not used in the substrate mixture. The activity of LOX was determined using sodium linoleate as the substrate in the reaction mixture.

#### 4.8.5. Determining TSP and LTGA Derivatives

Leaf tissue (100 mg) was ground as described above and homogenized in 1 mL of 80% (*v*/*v*) methanol solution. The extract was homogenized in a Thermomix at 25 °C for 12 h and the mixture was centrifuged at 13,000× *g* for 30 min. The methanolic extract was used to determine the TSP concentration and the pellet was kept at 20 °C to determine the concentration of LTGA derivatives according to Fortunato et al. [66].

#### 4.8.6. Determining Antioxidant Enzyme Activities

Leaf tissue (100 mg) was ground as described above and the fine powder was homogenized with 1 mL solution containing 100 mM potassium phosphate buffer (pH 7.8), 0.1 mM EDTA, 1 mM PMSF, and 0.5% (*w*/*v*) PVP. The homogenate was centrifuged at 13,000× *g* for 15 min at 4 °C and the supernatant was used to determine the activities of ascorbate peroxidase (APX) (EC 1.11.1.11), catalase (CAT) (EC 1.11.1.6), superoxide dismutase (SOD) (EC 1.15.1.1), and glutathione reductase (GR) (EC 1.8.1.7) following the procedures described by Debona et al. [67]. Briefly, APX activity was measured by the rate of ascorbate oxidation at 290 nm for 1 min at 25 °C. The CAT activity was determined by the rate of H_2_O_2_ decomposition at 240 nm for 1 min at 25 °C. The SOD activity was determined by measuring its ability to photochemically reduce the *p*-nitrotetrazole blue in a reaction mixture containing methionine and riboflavin. The production of formazan blue, which resulted from the photoreduction of *p*-nitrotetrazole blue, was measured at 560 nm. The GST activity was determined using reduced glutathione in the reaction mixture that contained 1-chloro-2,4-dinitrobenzene with absorbance measured at 340 nm.

### 4.9. Experimental Design and Data Analysis

A 2 × 2 factorial experiment, consisting of −Si and +Si plants that were non-inoculated (NI) or inoculated (I) with *B. maydis*, was arranged in a completely randomized design with four replications per each evaluation time to assess disease severity as well to determine the foliar concentrations of Si and K. Another 2 × 2 factorial experiment with the same factors mentioned above and six replications was carried out to evaluate the parameters of leaf gas exchange and Chl *a* fluorescence as well to quantify the foliar concentration of pigments. Leaf samples for the biochemical assays were obtained from another 2 × 2 factorial experiment with the same factors described above and five replications. Each experimental unit consisted of one plastic pot with two plants. All experiments were carried out twice. Data from variables and parameters were checked for normality and homogeneity of variance and subjected to analysis of variance. The treatment means were compared by the *F* test (*p* ≤ 0.05). The Minitab Statistical software was used for the statistical analysis mentioned above (Minitab, Inc., State College, PA, USA).

## Figures and Tables

**Table 1:** Analysis of variance for the effects of silicon rates (Si), plant inoculation (PI), and the Si × PI interaction for the variables and parameters evaluated.

Variables/Parameters *	Si	**PI**	**Si × PI**
Si	**<0.001**	0.244	0.901
K	0.185	0.222	0.634
Sev	**<0.001**	-	-
*A*	**0.0297**	**<0.001**	0.075
*g*_s_	0.093	0.709	0.502
*C*_i_	0.653	0.083	0.749
*E*	0.117	0.159	0.122
*F*_v_/*F*_m_	**0.002**	**<0.001**	**0.004**
Y(II)	**0.008**	0.258	0.377
Y(NPQ)	0.641	**0.005**	0.461
Y(NO)	0.813	0.718	0.799
ETR	**0.003**	0.06	0.095
Chl *a*+*b*	0.724	**<0.001**	0.374
Car	0.894	**0.008**	0.593
Glucose	0.124	**<0.001**	0.811
Fructose	0.229	0.205	0.485
Sucrose	0.067	**0.001**	**0.028**
Starch	0.345	**<0.001**	0.585
MDA	**<0.001**	**<0.001**	**0.001**
H_2_O_2_	0.293	0.082	0.198
O_2_^•−^	**0.001**	**<0.001**	**0.001**
TSP	0.783	**0.039**	0.375
LTGA derivatives	**0.005**	**<0.001**	**<0.001**
CHI	0.363	**0.015**	0.203
GLU	0.958	**0.008**	0.941
PAL	**0.040**	**<0.001**	0.166
PPO	0.463	**<0.001**	0.944
POX	0.569	**<0.001**	0.476
LOX	0.915	**<0.001**	0.845
SOD	0.110	**<0.001**	0.062
APX	0.128	**<0.001**	0.053
CAT	0.405	**<0.001**	0.568
GR	0.952	0.492	0.908

* Abbreviations: foliar concentrations of silicon (Si) and potassium (K), maydis leaf blight severity (Sev), leaf gas exchange parameters [rate of net CO_2_ assimilation (*A*), stomatal conductance to water vapor (*g*_s_), internal CO_2_ concentration (*C*_i_), and transpiration rate (*E*)], chlorophyll a fluorescence parameters [variable-to-maximum chlorophyll a fluorescence ratio (*F*_v_/*F*_m_), photochemical yield (Y(II)), yield for dissipation by downregulation (Y(NPQ), yield for non-regulated dissipation (Y(NO), and electron transport rate (ETR)], concentrations of photosynthetic pigments [chlorophyll a+b (Chl *a*+*b*) and carotenoids (Car)], carbohydrates (glucose, fructose, sucrose, and starch), malondialdehyde (MDA), hydrogen peroxide (H_2_O_2_), and superoxide anion radical (O_2_^•−^), total soluble phenolics (TSP) and lignin-thioglycolic acid (LTGA) derivatives, as well as activities of defense [chitinase (CHI), β-1,3-glucanase (GLU), phenylalanine ammonia-lyase (PAL), polyphenoloxidase (PPO), peroxidase (POX), and lipoxygenase (LOX)] and antioxidative [superoxide dismutase (SOD), ascorbate peroxidase (APX), catalase (CAT), glutathione reductase (GR)] enzymes. Bold values are significant at *p* ≤ 0.05.

## Data Availability

The datasets supporting the conclusions of this article are included within the article.
